# Mapping sites of herpes simplex virus type 1 glycoprotein D that permit insertions and impact gD and gB receptors usage

**DOI:** 10.1038/srep43712

**Published:** 2017-03-03

**Authors:** Qing Fan, Sarah Kopp, Sarah A. Connolly, William J. Muller, Richard Longnecker

**Affiliations:** 1 Department of Microbiology–Immunology, Northwestern University Feinberg School of Medicine, Chicago, Illinois, USA; 2 Department of Health Sciences, Department of Biological Sciences, DePaul University, Chicago, Illinois, USA; 3 Pediatrics, Northwestern University Feinberg School of Medicine, Chicago, Illinois, USA

## Abstract

Glycoprotein D (gD) of herpes simplex virus type 1 (HSV-1) is one of four glycoproteins essential for HSV entry and cell fusion. The purpose of this study was to determine the plasticity of gD to tolerate insertion or deletion mutations and to construct an oncolytic HSV-1 that utilizes the disialoganglioside GD2 as a HSV-1 entry receptor. We found that the N-terminus of gD tolerates long insertions, whereas residues adjacent to the gD Ig-like V-type core tolerated shorter insertions (up to 15 amino acids), but not greater than 60 amino acids. Recombinant HSV-1 containing the ch14.18 single chain variable fragment (scFv) at the N-terminus of gD failed to mediate entry, even though the ch14.18 scFv-gD chimera Fc bound to neuroblastoma cells expressing GD2. Finally, we found that hyperfusogenic gB mutants enhanced fusion to a greater degree with the gB receptor the paired immunoglobulin-like type 2 receptor alpha (PILRα) than with gD receptors HVEM and nectin-1. Hyperfusogenic gB could restore the fusion function with PILRα when a gD constructed contained only the “profusion domain” (PFD), suggesting the hyperfusogenic form of gB may regulate fusion of PILRα via a novel mechanism through gH/gL and the gD PFD.

Herpes simplex virus type 1 (HSV-1) is an alphaherpesvirus that typically causes mucocutaneous lesions on the mouth and occasionally causes severe disease such as meningitis or encephalitis. Four viral glycoproteins (gD, gB, gH, and gL) are required for the entry of HSV-1 into cells. Glycoprotein D (gD) is the receptor binding protein and its binding to receptor is proposed to activate the heterodimer gH/gL to trigger gB to mediate the fusion of viral and cellular membranes^1^,^2^. Receptors for HSV-1 gD include nectin-1^3^,^4^,^5^,^6^,^7^, herpes virus entry mediator (HVEM)^8^, and a modified form of heparan sulfate^9^,^10^. The paired immunoglobulin-like type 2 receptor alpha (PILRα), a receptor that binds to HSV-1 gB, also has been reported to mediate HSV-1 fusion^11^.

Previous mutagenesis and structural studies of gD have proposed a model in which the C-terminus of the gD ectodomain folds back on itself to inhibit the receptor-binding site on gD^12^. Upon HSV-1 binding to target cells, receptor binding to gD displaces the C-terminus of the gD ectodomain and this conformational change may signal the other glycoproteins to mediate fusion. The infectivity of a HSV-1 lacking gD can be restored by the addition of exogenous soluble gD, but only if the C-terminal residues of the gD ectodomain (P261-P305) are present in that soluble form. Thus, the C-terminal region of the gD ectodomain has been termed the gD profusion domain (PFD)^13^. Most of the proline-rich PFD is not resolved in the gD crystal structures^12^,^14^,^15^. Coimmunoprecipitation experiments suggest that gH/gL and gB may interact with the gD PFD^16^. Previously, we demonstrated a functional interaction between gD and gH/gL using the entry glycoproteins of the primate saimiriine herpesvirus 1 (SaHV-1)^17^,^18^.

The original aim of our current studies was to retarget HSV-1 to neuroblastoma as a therapeutic option for this cancer. Neuroblastoma is the most common cancer in infancy and the most common extracranial solid cancer in childhood^19^. HSV-1 gD has been used for cell tropism retargeting by constructing recombinant HSV in which gD is fused to a heterologous ligand that can interact with the tumor‐specific receptor of choice. Using this approach, HSV-1 has been retargeted to the IL13Rα2 receptor^20^, the human epidermal growth factor receptor 2 (HER-2)^21^,^22^, the urokinase plasminogen activator (uPA) receptor^23^, epithelial cell adhesion molecule (EpCAM)^24^, human epidermal growth factor receptor (EGFR)^25^, or human carcinoembryonic antigen (CEA)^25^. Building on these studies, we selected a heterologous ligand the single chain variable fragment (scFv) from the monoclonal antibody (MAb) chimeric 14.18 (ch14.18, kindly provided by EMD Serono Research & Development Institute, Inc.) that binds to neuroblastoma cells expressing GD2^26^. GD2 is a disialoganglioside displayed on the surface of cells of neuroectodermal origin, which is present at high amounts on neuroblastoma and other types of cancer cells but at much lower levels on normal tissue^27^. ch14.18 is a humanized chimeric anti-GD2 monoclonal antibody derived from a murine monoclonal raised against a neuroblastoma cell line^27^. It has complement- and antibody-dependent cytoxic activity^27^, and it is used clinically for treatment of high-risk neuroblastoma^28^, and has also been shown to function in the context of a chimeric receptor^29^. Given the activity of ch14.18 against cells expressing GD2, we wanted to determine if ch14.18 scFv might serve as a tool to create an oncolytic HSV-1 for treating neuroblastoma, including investigating if the virus could be retargeted to infect neuroblastoma cells in the absence of a gD interaction with HSV-1 receptors, the functional interaction that triggers wild-type HSV fusion.

To achieve this goal, we first sought to perform a careful evaluation of which regions of gD would tolerate large insertions by testing the cell surface expression and cell fusion of a collection of gD insertion mutants. Previously, linker insertion mutagenesis of gD showed that gD can tolerate short insertions (5 amino acids (aa)) at some sites on gD without abrogating fusion with HVEM and nectin-1^30^. Based on this study, we selected insertion sites at gD residues at 12, 34, 187, and 243. In addition, we selected an insertion site at residue 260, immediately upstream of the PFD of gD. We generated a panel of gD mutants by inserting different lengths of protein domains with known or predicted structural characteristics, including the 289 aa ch14.18 scFv. We investigated the function of the gD profusion domain (PFD) when coexpressed with a hyperfusogenic variant of gB. The use of hyperfusogenic forms of gB may have utility in the design of oncolytic HSV vectors.

## Results

### Mutations in gD affect cell surface expression

**Figure 1:**
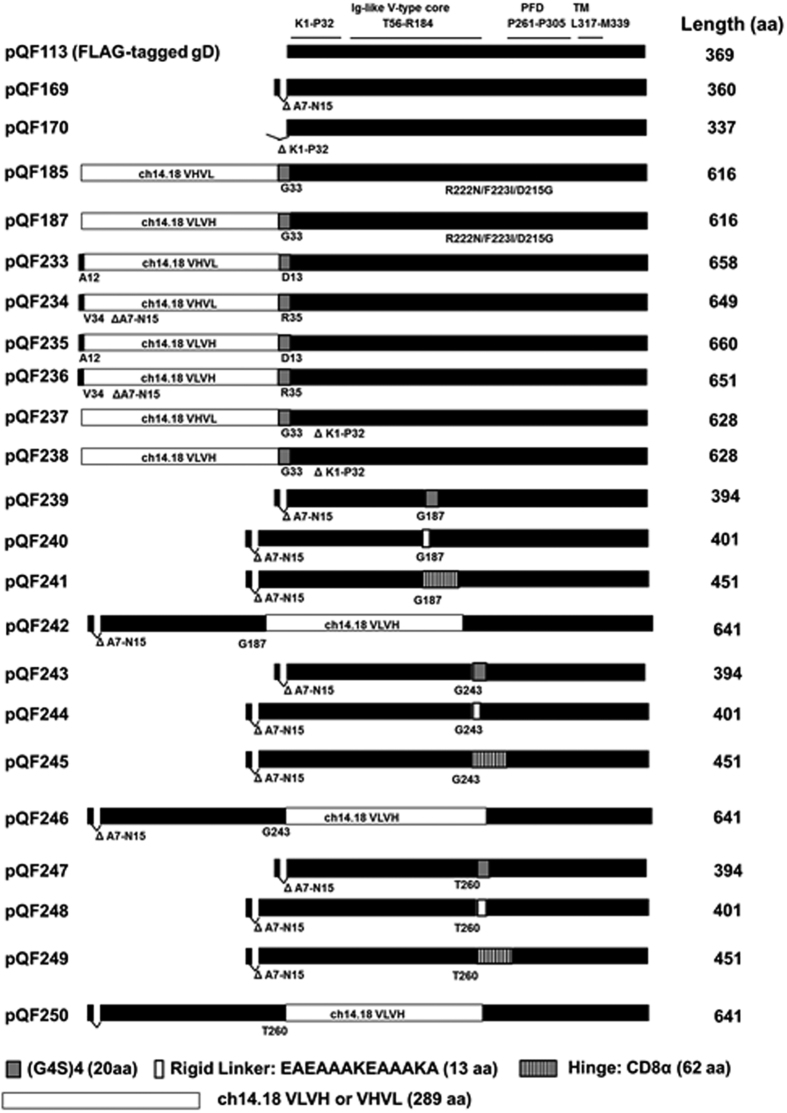
Schematic representation of the gD constructs. Black bars represent HSV-1 gD sequence. For all constructs (except pQF185 and pQF187, which were cloned into pCAGGS), the native signal sequence was replaced by an exogenous signal sequence and an N-terminal FLAG tag, which is not shown in the diagram. The amino acids were numbered after signal peptide cleavage sites according to WT gD (369 aa). K1-P32 is the HVEM binding region. T56-R184 is HSV-1 gD core that adopts an Ig-like V-type fold. P261-P305 is the profusion domain (PFD). L317-M339 is the transmembrane domain (TM). Y369 is the last amino acid of the gD protein. Five protein domains (a 289 aa ch14.18 scFv VLVH, a 289 aa ch14.18 scFv VHVL, a 62 aa CD8α flexible hinge, a 13 aa rigid linker EAEAAAKEAAAKA and a 20 aa flexible (GGGGS)4 were used to replace the gD sequence or to insert at different residues of gD. The total length in aa of each construct was shown in the figure.

In order to investigate the plasticity of gD, a collection of gD mutants with various deletions and small and large insertions was created (Fig. 1, Table 1). With the exception for pQF185 and pQF187, the HSV-1 gD native signal sequence was replaced in all constructs with a heterologous signal sequence containing a N-terminal FLAG epitope. The FLAG tagged gD (F-gD) was then mutated by deletion and/or insertion. The plasmids pQF169 and pQF170 were generated by deleting A7-N15 and K1-P32 from F-gD^17^, respectively (Fig. 1, Table 1). These deletions eliminate the HVEM receptor binding site and these plasmids serve as the basis for the additional gD constructs that were generated^31^. To introduce a large insertion into gD, the 289 aa single chain-chain variable antibody domain (scFv VLVH or VHVL) from the antibody ch14.18 that recognizes the disialoganglioside GD2^26^ was used. For a medium length insertion, we chose to use the 62 aa CD8α molecule hinge domain^32^. Finally, for short insertions, we used the 20 aa flexible protein linker (G4S)4 or a 13 aa short rigid protein linker (EAEAAAKEAAAKA). The longest insertion of 289 aa scFv VLVH or VHVL resulted in the larger constructs (pQF185, pQF187, pQF233-pQF238, pQF242, pQF246 and pQF250), with total length in amino acid between 616 aa-658 aa (Fig. 1).

**Figure 2:**
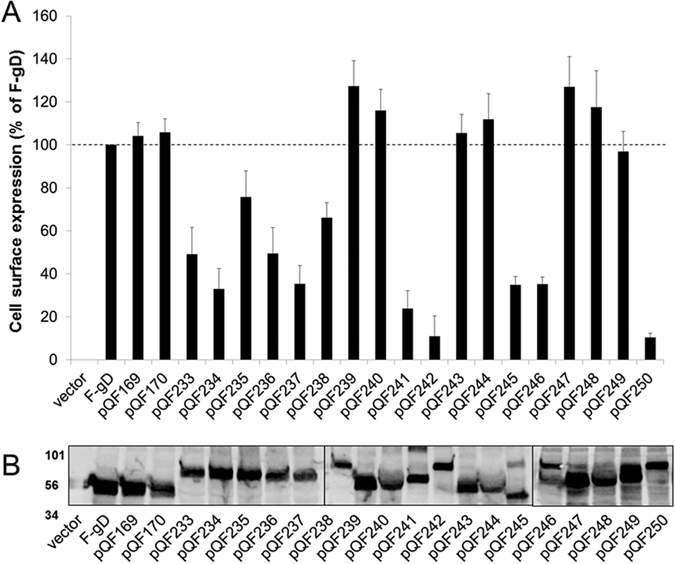
Expression of gD mutants. (**A**) Cell surface expression measured by CELISA. CHO-K1 cells in a 96-well plate were transfected overnight with plasmids encoding FLAG-tagged gD (F-gD), gD mutants, or an empty vector. The cells were washed and incubated with an anti-FLAG M2 antibody. After extensive washing, cells were fixed and incubated with an anti-mouse secondary antibody for detection. Each bar shows the mean and standard deviation of three independent determinations. Background signals (vector control) were subtracted from the values. Data for each set of glycoproteins were normalized to the expression level of F-gD. (**B**) Total protein expression measured by western blot of cell lysates. CHO cells expressing the constructs above were lysed and proteins were resolved by SDS-PAGE. Proteins were transferred to nitrocellulose and probed with rabbit anti-FLAG antibody followed by goat anti-rabbit IgG. All mutants migrated to their predicted molecular weights.

The cell surface expression of the chimeric glycoproteins was examined by CELISA by transfecting CHO-K1 cells with the constructs and probing for the FLAG epitope^33^,^34^. For some of the gD mutants (pQF169-170, pQF239-240, pQF243-244, and pQF 247-249), gD expression was similar to WT F-gD (Fig. 2A). Most of these mutants had short insertions (13 or 20 aa), indicating that short insertions did not alter cell surface expression. Only one mutant, pQF249 with 62aa inserted at residue 260, was expressed at wild-type levels. The position of this 62 aa insertion was critical, since this insertion was not tolerated as well at residue 187 (pQF241) or 243 (pQF245). Several mutants, including pQF233-238 and pQF245-246, showed reduced cell surface expression (30-50% of the level of F-gD). pQF241, pQF242 and pQF250 had the lowest cell surface expression, suggesting that these mutants were not appropriately processed and transported to the cell surface. A western blot of total cell lysate confirmed expression of the gD mutants and each mutant tested migrated at the expected molecular weight (Fig. 2B).

### N-terminal deletions in gD alter receptor usage in cell-cell fusion

**Figure 3:**
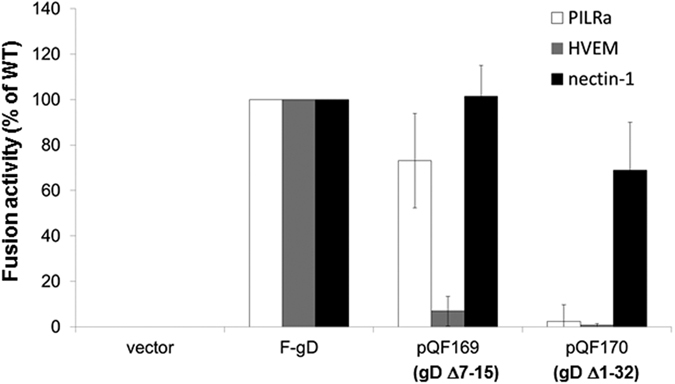
Cell fusion activity of HSV-1 gD N-terminal deletion mutants. Target cells were transfected with a reporter plasmid encoding luciferase under control of the T7 promoter along with plasmids encoding PILRα, HVEM, or nectin-1. The transfected cells were replated with effector cells that had been transfected with plasmids encoding T7 polymerase, gB, gH, gL, and either F-gD, pQF169, pQF170, or vector. After co-incubation overnight, luciferase activity was measured as an indication of cell-cell fusion. For each set of viral glycoproteins, data were normalized to the fusion activity separately for each receptor mediated by F-gD, after subtraction of background signals (vector control). Each bar shows the mean and standard deviation of at least three independent determinations.

A quantitative cell-cell fusion assay was used to assess cell fusion activity of these mutants using target cells overexpressing the gB receptor PILRα or the gD receptors nectin-1 and HVEM (Fig. 3). As expected, pQF169 and pQF170 did not use HVEM as a fusion receptor the HVEM binding domain in gD was deleted (∆7–15 for pQF169 and ∆1–32 for pQF170). pQF170 abrogated the fusion with HVEM completely while pQF169 only showed 6% fusion activity of the F-gD, consistent with previous results^31^. Both pQF169 and pQF170 retained robust fusion activity with nectin-1. Most interestingly, pQF170 abrogated the usage of PILRα as a fusion receptor, but pQF169 maintained fusion with PILRα (Fig. 3), suggesting the first 32 aa of gD may contribute to PILRα function as a gB fusion receptor. This result supports the idea that function of PILRα requires an endogenous gD receptor present in CHO cells^11^,^35^.

### The gD insertion mutations alter receptor usage in cell-cell fusion

**Figure 4:**
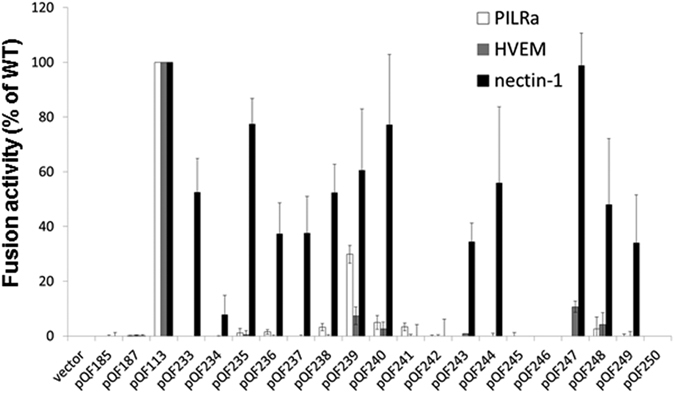
Cell fusion activity of HSV-1 gD mutants. Target cells were transfected with a reporter plasmid encoding luciferase under control of the T7 promoter along with plasmids encoding PILRα, HVEM and nectin-1. The transfected cells were replated with effector cells that had been transfected with plasmids encoding T7 polymerase, gB, gH, gL and either F-gD, a gD mutant, or vector. After co-incubation overnight, luciferase activity was measured as an indication of cell-cell fusion. For each set of viral glycoproteins, data were normalized to the fusion activity separately for each receptor mediated by F-gD, after subtraction of background signals (vector control). Each bar shows the mean and standard deviation of at least three independent determinations.

The quantitative cell-cell fusion assay was used to examine the cell fusion activity of the gD mutants. As expected, most gD mutants did not function with HVEM (Fig. 4) because most of the constructs were deleted for the HVEM binding site (Fig. 1, Table 1). Interestingly, mutants carrying a 20 aa insertion at residue 187 (pQF239) or 260 (pQF247) showed 5–10% fusion activity with HVEM, similar to the low level of fusion with HVEM observed for the parental construct pQF169 (Figs 3 and 4).

Mutations of gD showed quite different fusion activity when the target cells overexpressed nectin-1. As expected, pQF185 and pQF187 showed no fusion because these constructs include three mutations that abrogate nectin-1 mediated fusion (pDM80)^36^. Constructs with insertions of the long VHVL or VLVH sequences (289 aa) at the gD N-terminus (pQF233-238) maintained 38–80% fusion activity with nectin-1, with the exception of pQF234. Among the constructs with ch14.18 scFV insertions, a VLVH insertion at residue 12 with a G4S(4) linker (pQF235) showed the greatest fusion activity (Fig. 4).

All of the constructs with short insertions (pQF239-240, pQF243-244, pQF247-249) showed fusion activity with nectin-1 (34–98%). Insertion of (G4S)4 at residue 260 (pQF247) resulted in wild-type levels of fusion activity with nectin-1. These results suggest that gD tolerates insertions of greater than 5 aa, as showed previously^30^, including a 20 aa flexible linker (G4S)4 or a 13 aa rigid linker (EAEAAAKEAAAKA) at resdiues G187, G243 and T260 (Fig. 4). Insertion of a flexible (G4S)4 linker (pQF243) versus a rigid linker (pQF244) at G243 did not alter fusion activity greatly. At residue G187, insertion of rigid linker (pQF240) showed higher fusion with nectin-1 than insertion of the (G4S)4 linker (pQF239). The opposite effect was seen for insertions at T260 (pQF247 vs. pQF248) (Fig. 4).

Insertion of the 62 aa CD8α hinge at G187 (pQF241) and G243 (pQF245) completely abrogated the fusion with nectin-1. In contrast, insertion of the CD8α hinge at T260 (pQF249) reduced but did not eliminate function with nectin-1, indicating that the sequence before T260 contributes to nectin-1 activity. Insertion of the VLVH together with the linker (G4S)4 domain at G187, G243 and T260 resulted in lower cell surface expression (Fig. 2) and a complete abrogation of the cell fusion, suggesting that, although some positions can tolerate shorter insertions, longer insertions at these sites are not compatible with fusion function. This may be due to the insertions changing the overall gD conformation or blocking the interaction of gD with nectin-1.

Mutations in gD also affected the fusion function of PILRα. Although F-gD and pQF169 were functional with PILRα (Fig. 3), most of the gD insertions created in this study completely abrogated fusion with PILRα (Fig. 4). The only gD construct that retained function with PILRα was pQF239, which has a small 20 aa insertion of (G4S)4 at residue 243.

### Hyperfusogenic gB mutants promote greater fusion with PILRα, even in the absence of HSV-1 gD

**Figure 5:**
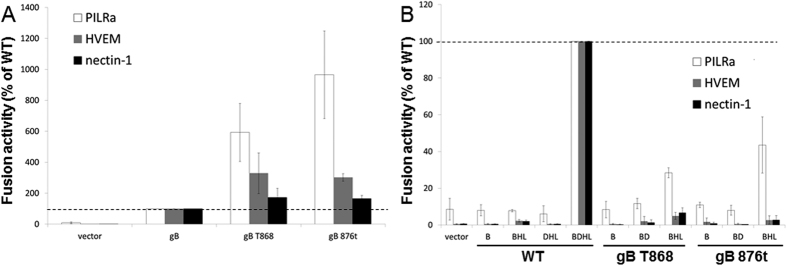
Cell fusion activity of hyperfusogenic gB mutants. Target cells were transfected with a reporter plasmid encoding luciferase under control of the T7 promoter along with plasmids encoding PILRα, HVEM or nectin-1. (**A**) The transfected cells were replated with effector cells that had been transfected with plasmids encoding T7 polymerase, gH, gL, gD, and either WT gB, hyperfusogenic gB, or vector. After co-incubation overnight, luciferase activity was measured as an indication of cell-cell fusion. (**B**) The fusion assay was performed as above, but the effector cells were transfected with plasmids encoding T7 polymerase and combinations of WT gD, gH, gL, and/or vector, along with either WT gB or hyperfusogenic gB. For each set of viral glycoproteins, data were normalized to the fusion activity separately for each receptor mediated by WT gB, gD, gH/gL, after subtraction of background signals (vector control). Each bar shows the mean and standard deviation of at least three independent determinations.

Previous studies had found that the HSV-1 gB receptor PILRα needs gD and a gD receptor to mediate fusion^11^,^35^. The interaction of gB with its receptor PILRα requires the presence of sialylated O*-*glycans on gB. Two threonine residues in gB are essential for the addition of the O*-*glycans that are required for gB binding to PILRα^37^. To investigate if hyperfusogenic forms of gB enhance fusion when PILRα is used as an entry receptor for HSV-1, we used gB T868^33^ and gB 876t hyperfusogenic gB mutants that carry a cytoplasmic tail truncation^38^,^39^. Using the quantitative cell-cell fusion assay, hyperfusogenic gB mutants showed enhanced fusion with target cells expressing HVEM, nectin-1, or PILRα (Fig. 5A). Most interestingly, the gB hyperfusogenic mutations impacted fusion with PILRα expressing cells to the greatest degree, with the fusion activity enhanced 6-fold for T868 and 10-fold for gB 876t compared to WT gB fusion levels.

To determine whether the hyperfusogenic gB mutants retained a requirement for the other glycoproteins, we expressed different combinations of HSV-1 entry glycoproteins in the effector cells. As expected, WT gB alone, gB with gH/gL, or gD with gH/gL did not induce fusion with any receptor (Fig. 5B). When gB T868 or gB 876t was expressed alone, or coexpressed with WT gD, fusion activity was barely detected with any receptor (Fig. 5B). Surprisingly, fusion activity was detected when effector cells expressing hyperfusogenic gB and gH/gL only were mixed with target cells expressing PILRα, but not HVEM or nectin-1. This indicates that the gB hyperfusogenic mutants function with WT gH/gL better than WT gB, at least in the absence of gD (Fig. 5B). The fusion of gB876t or gBT868 with gD, gH and gL was shown not in Fig. 5B but in Fig. 5A.

### The gD profusion domain functions with PILRα

**Figure 6:**
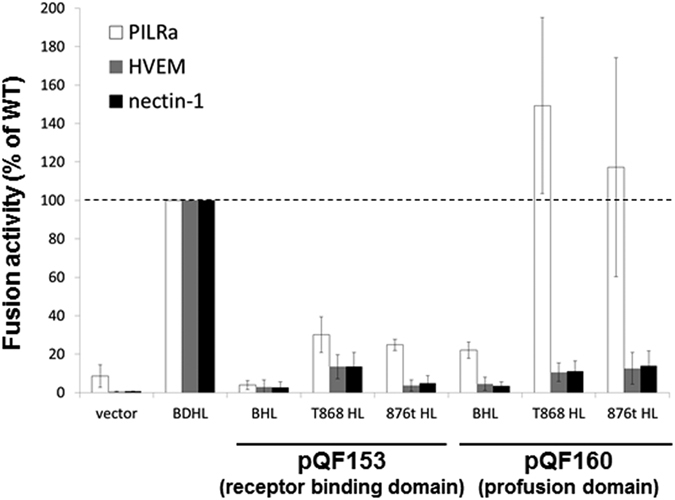
Cell fusion activity of HSV-1 gD profusion domain and hyperfusogenic gB. Target cells were transfected with a reporter plasmid encoding luciferase under control of the T7 promoter along with plasmids encoding PILRα, HVEM, or nectin-1. The transfected cells were replated with effector cells that had been transfected with plasmids encoding T7 polymerase and combinations of gH, gL WT gB or hyperfusogenic gB, and WT or mutant gD, and vector. After co-incubation overnight, luciferase activity was measured as an indication of cell-cell fusion. For each set of viral glycoproteins, data were normalized the fusion activity separately for each receptor mediated by WT gB, gD, gH/gL, after subtraction of background signals (vector control). Each bar shows the mean and standard deviation of at least three independent determinations. RBD: receptor binding domain. PFD: profusion domain.

To investigate the gD domains that function in fusion with the hyperfusogenic gB, we examined the cell fusion activity of two previously reported gD deletion mutants (pQF153 and pQF160)^17^. pQF153 is a construct that includes the gD receptor binding domain (RBD, K1-T160) plus the transmembrane domain and cytoplasmic tail (Y306-Y369). pQF160 is a gD construct in which the RBD has been deleted, such that it includes the only the gD profusion domain (PFD) along with the transmembrane domain and cytoplasmic tail (P261-Y369). The gD RBD construct (pQF153) did not show any fusion activity with HVEM, nectin-1, or PILRα when coexpressed with WT gB, and gH/gL (Fig. 6). Coexpression of hyperfusogenic gB with the gD RBD (pQF153) and gH/gL rescued a limited degree of fusion, but only with the PILRα receptor. The gD PFD construct (pQF160) mediated a modest degree of fusion activity when coexpressed with WT gB and gH/gL, however only with the PILRα receptor. Interestingly, coexpression of hyperfusogenic gB with the gD PFD construct (pQF160) and gH/gL completely restored fusion with PILRα, indicating that the gD PFD may play a larger role in fusion function than the receptor binding domain in the context of PILRα mediated fusion.

### Soluble scFV-gD bound to neuroblastoma cells whereas an HSV-1 recombinant containing the same soluble scFv-gD was unable to infect neuroblastoma cells expressing GD2

**Figure 7:**
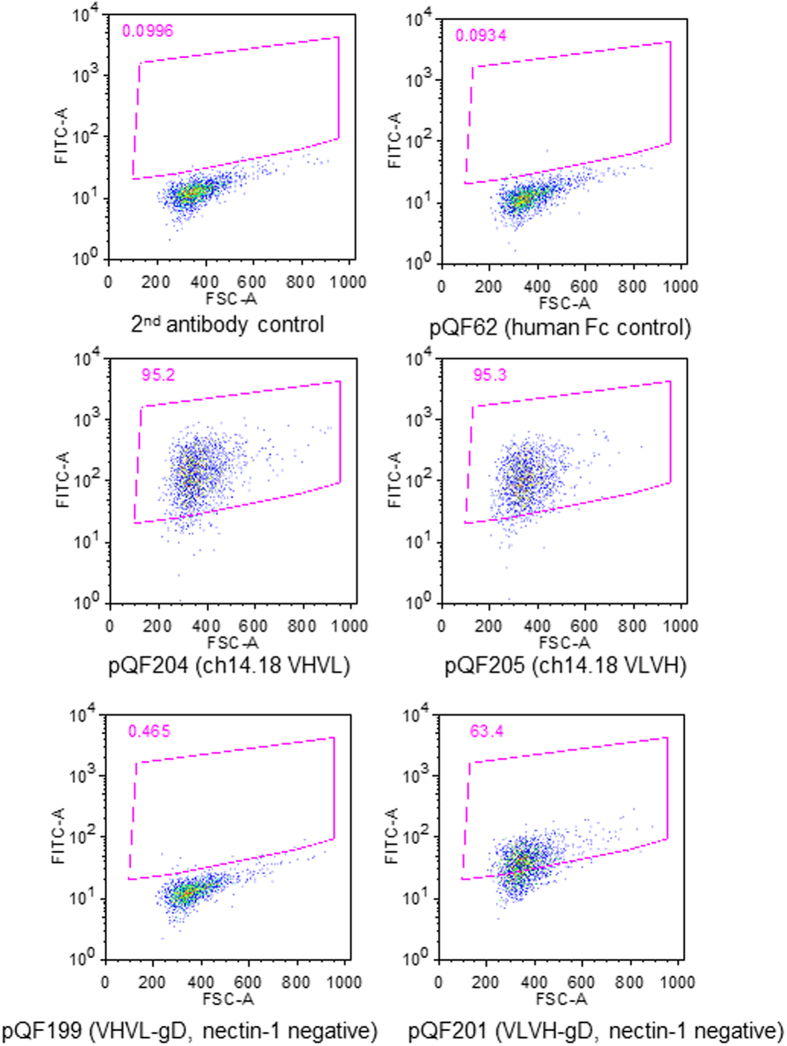
Binding of the scFv-gD chimeras to neuroblastoma cells. Neuroblastoma cells SK-N-Be (2) cells were stained with the normalized supernatants from cells that were transfected with pQF62 (human Fc control), pQF204 (ch14.18 scFV VHVL), pQF205 (ch14.18 scFv VLVH), pQF199 (gD linked to ch14.18 VHVL) or pQF201 (gD linked to ch14.18 VLVH). The nectin-1 binding on gD was mutated in both pQF199 and pQF201. Binding was detected by staining with the secondary anti-human IgG FITC.

We first examined whether the insertion of scFv into gD was properly folded by investigating the binding of soluble versions of scFv-gD to neuroblastoma cell lines expressing GD2. The gD transmembrane domain was deleted and the human Fc binding domain was added to the gD C-terminus to allow for easy detection of the binding to the neuroblastoma cell lines SK-N-Be (2), LAN5, and S5Y5. SK-N-Be (2) cells were incubated with supernatants from cells transfected with constructs encoding either human Fc (pQF62) as a control, Fc linked to ch14.18 scFv VHVL (pQF204) or VLVH (pQF205), or Fc linked to gD with ch14.18 scFv VHVL (pQF199) or VLVH (pQF201) at the gD N-terminus in addition to mutations that prevent nectin-1 or HVEM usage. As expected, the secondary antibody did not react with the SK-N-Be (2) cells (Fig. 7), LAN5 (data not shown), or S5Y5 cells (data not shown). The positive controls of ch14.18 scFv VHVL and VLVH fused with the Fc region (pQF204 and pQF205) bound well to SK-N-Be (2) cells. Interestingly, the gD VHVL fusion protein (pQF199) had no detectable binding to SK-N-Be (2) cells, whereas in the gD VLVH fusion protein (pQF201) bound to the SK-N-Be (2) cells well (Fig. 7), indicating the orientation of the scFv in gD affected the ability of the scFv to bind to GD2.

Based on these binding data, we generated recombinant viruses introducing gD with ch14.18 scFv VLVH at the gD N-terminus with or without mutations that prevent nectin1 and HVEM usage (pQF187 and pQF238, respectively). These gD constructs were cloned into an RFP containing HSV-1 BAC. pQF187 and pQF238 have the first 32 aa of N terminal gD domain replaced with VLVH of ch14.18 scFv, the GD2-specific MAb. pQF187 was made from pDM80, a gD construct with three mutations that abrogate gD nectin-1 binding, whereas pQF238 has an intact nectin-1 binding site. As expected, pQF238 mediated fusion with nectin-1 expressing cells but not HVEM expressing cells, whereas pQF187 does not function with either gD receptor (Fig. 4). The resulting BACs, as well as control BAC made based on pQF238, were transfected into SK-N-Be (2), LAN5, and S5Y5 neuroblastoma cell lines, as well as WT gD-expressing VD60 cells. Despite observing abundant single RFP expressing cells, we detected no spread to adjacent cells. In WT BAC (GS3217) transfections, abundant cytopathic effect was readily observed in the neuroblastoma cells (data not shown). The reason for the lack of infectivity scFv containing BACs is not clear. When the same BACs were transfected into gD expressing cells VD60, virus was produced (data not shown), indicating that the lack of growth was due to the lack of a functional gD and not to a spurious mutation acquired by the BAC manipulation in bacteria. Since the chimeric VLVH gD (pQF201) was able to bind to cells, the observed defect in virus replication may be due to changes in avidity of binding to receptor or altering the confirmation of gD so that it is no longer functional to trigger fusion, even though pQF238 was able to mediate fusion with nectin-1 expressing cells in the cell fusion assay (Fig. 4). The defect may be due to an inability of GD2 to function as a receptor that can trigger fusion.

## Discussion

The present study was initiated to investigate regions of HSV-1 gD that tolerate insertion of a heterologous protein sequence and preserve gD fusion function. The work was intended to inform the development of an oncolytic HSV-1 vector that could detarget gD from its natural receptor and retarget HSV-1 to GD2 expressed on neuroblastoma cells. To begin the studies, we investigated the plasticity of gD to tolerate insertion of various protein domains including a flexible linker (G4S)4, a rigid linker (EAEAAAKEAAAKA), a flexible spacer CD8α hinge, and finally a single-chain variable antibody fragment (scFv). Since the majority of the constructs used in this study were based on constructs in which the HVEM binding site was disrupted or included insertions adjacent to the HVEM binding site, the failure of these constructs to mediate fusion with HVEM-expressing cells was expected (Figs 3 and 4). We mainly focused another gD receptor nectin-1, a pan receptor for alphaherpesviruses^3^,^4^,^17^,^40^,^41^.

The fusion activity of the gD constructs indicated that the N-terminus of gD tolerates longer insertions, whereas the residues within the gD ectodomain tolerate 15 aa insertions but not 60 aa insertions (Fig. 4). Cell fusion with nectin-1 was largely dependent on the length of the insertion and not the specific rigid or flexible linker (Fig. 4).

Even though the O-glycosylation on HSV-1 gB mediate interaction with PILRα^37^, fusion mediated by PILRα still requires gD and a gD receptor^11^,^35^. We used our panel of gD mutants to examine how gD impacts fusion with PILRα expressing cells. Interestingly, fusion activity of most gD mutants with PILRα as a receptor was abrogated or greatly reduced, even though some of these mutants retained functioned with nectin-1 (Figs 3 and 4). Deletion of aa 7–15 (pQF169) did not prevent function with PILRα, however, fusion with PILRα was greatly reduced when gD aa 1–32 were deleted (pQF170) (Fig. 3). pDM80, the triple mutant of gD that does not mediate fusion with nectin-1, also showed a significantly reduced fusion with PILRα (data not shown). The impact of the gD mutations on fusion with PILRα expressing cells may be the result of the gD mutants losing binding to an endogenous gD receptor present in CHO cells^35^. The fact that pQF169 (which cannot bind to HVEM) retains fusion function on PILRα expressing cells support the idea that the endogenous receptor on CHO cells may be a nectin-1-like receptor.

Although WT fusion requires all four HSV entry glycoproteins, studies exploring the functional interactions among the glycoproteins have demonstrated that mutations in several proteins can overcome this strict interdependence. When the gH N-terminus is deleted (gHΔ48), this mutant gH/gL can induce low levels of fusion by gB in the absence of wild type gD and/or receptor^42^. A gD-V231W mutant also can induce cell-cell fusion in the absence of receptor^43^. The gD-V231W is proposed to displace the gD C terminus in a manner that mimics the conformational change in gD that occurs after receptor binding. The receptor-binding and fusion-activating functions of gD can also be replaced by scFv, as has been shown in recombinant viruses. A scFv that binds to the cancer-specific HER2 receptor has been added to gH^44^ and fusion proceeds. Two gB syncytial mutants, gB A855V and A874P, can trigger low levels of cell-cell fusion with HVEM in the absence of either gD or gH/gL^45^. The site of physical interaction with gH/gL on gD may map to the PFD or the PFD may support a gH/gL inetraction site on gD. Our previous study showed that the gD PFD sequence contributes to the homotypic interaction observed used HSV-1 and SaHV-1 chimeras^17^.

The biochemical properties of the C-terminal cytoplasmic domain of gB have been extensively studied and syncytial mutants are readily obtained such as truncations at gB876 and gB868^39^. Interestingly, we found that these mutants were hyperfusogenic not only with HVEM and nectin-1, but also with PILRα (Fig. 5). When the gD profusion domain alone (pQF160) was coexpressed with hyperfusogenic gB and gH/gL, fusion was significantly increased for PILRα, but not HVEM and nectin-1 (Fig. 6). Future studies will focus on how the gD PFD allows the bypass of the interaction of gD with a gD receptor when the target cells express PILRα, as well as the interaction of hyperfusogenic gB and gD or gH/gL.

Oncolytic HSV can be generated by modifying the virus genetically or by retargeting the virus from its natural receptor. Talimogene laherparepvec (T-VEC) is a genetically engineered HSV-1 strain created after a multi-step process which both attenuated the virus and promoted replication in tumor cells but not normal cells. T-VEC is armed with granulocyte macrophage colony-stimulating factor (GM-CSF), and it is the first oncolytic virus immunotherapy approved by the US Food and Drug Administration (FDA) for the treatment of melanoma^46^. The success of T-VEC encourages more effort to develop oncolytic HSV-1 to treat different cancers.

HSV-1 gD can be retargeted to a variety of different tumor antigens redirecting HSV-1 entry and resulting in oncolytic viruses for the potential use in cancer treatment^20^,^21^,^22^,^23^. Our attempts in the current study to retarget gD to use GD2 as a receptor were similar to recent reports detailing the retargeting of HSV to human epidermal growth factor receptor (EGFR) and the human carcinoembryonic antigen (CEA)^25^, or the cellular epithelial cell adhesion molecule (EpCAM)^24^. The difference is that GD2 is a disialoganglioside, while EGFR, CEA and EpCAM are all membrane proteins. Even though we were unable to demonstrate fusion of GD2-expressing cells mediated by the ch14.18 scFv gD mutants pQF187 or pQF238, our work provides evidence that insertion of longer protein sequences at different positions of the N-terminus of gD does not abrogate fusion with nectin-1 (Fig. 4). Further studies will be needed to determine whether scFv for other neuroblastoma cancer markers, like CD133 (prominin-1)^47^, or helicase DNA-binding protein 5 (CHD5)^47^,^48^ can be used to alter HSV tropism and allow targeting of neuroblastoma.

## Materials and Methods

### Cells

Chinese hamster ovary (CHO-K1, ATCC) cells were maintained in Ham’s F12 medium supplemented with 10% fetal bovine serum (FBS). 293 PEAK Rapid cells (Edge Biosystems) were grown in DMEM supplemented with 10% FBS. The neuroblastoma cell lines SK-N-BE(2), LAN-5, and S5Y5 were kindly provided by Children’s Oncology Group Cell Culture and Xenograft Repository). VD60 cells, which express gD, were used to complement gD negative viruses as previously described^49^. SK-N-Be(2), S5Y5 and VD60 were grown in Dulbecco’s modified Eagle’s medium supplemented with 10% heat inactivated fetal calf serum. LAN-5 was grown in RPMI medium supplemented with 10% FBS.

### Plasmids and BACs

Plasmids encoding HSV-1 (KOS) gB (pPEP98), gD (pPEP99), gH (pPEP100) and gL (pPEP101) were described previously^50^, as were plasmids encoding human nectin-1 (pBG38)^4^, human HVEM (pBEC10)^8^, and human PILRα (pQF003)^35^. pQF113 (named as F-gD in this paper) contains wild type HSV-1 gD without its native signal peptide cloned in pFLAG-myc-CMV-21 expression vector (E5776; Sigma)^17^, pDM80 is a triple gD mutant (R222N/F223I/D215G) subcloned into pcDNA3^36^. gB T868 is an insertion gB mutant with 5 aa linker (SCLNT) at residue 868^33^ and gB 876t^38^ with final 28 residues of the wild-type gB tail truncated. Both mutations had been shown to be hyperfusogenic^38^. pQF153 and pQF160 are gD deletion mutants which have the PFD or receptor binding dormain deleted from gD^17^. pQF169 and pQF170 were generated by deleting A7-N15 and K1-P32 from F-gD (pQF113)^17^, respectively. Plasmids pdCs 14.18 VHVL-Fc and pdCs 14.18 VLVH-Fc were provided by EMD Serono Research & Development Institute, Inc. Table 1 and Fig. 1 depict the constructs created for this study. Constructs pQF233 through pQF250 were generated by subcloning HSV-1 glycoproteins gD mutations into pFLAG-myc-CMV-21 expression vector downstream of the FLAG epitope with the native gD signal sequences removed. pQF185 and pQF187 were cloned by replacing gD aa K1-P32 in PDM80 with the VHVL and VLVH from pdCs 14.18 VHVL-Fc and pdCs 14.18 VLVH-Fc, respectively. pQF62^51^ is a construct containing the human Fc and was used as a control in this study. To generate Fc tagged soluble variants of gD with the VHVL and VLVH GD2 binding regions, the gD ectodomains from pQF185 and pQF187 were cloned into pQF62 generating pQF199 and pQF201, respectively. VHVL or VLVH from pdCs 14.18 VHVL-Fc and pdCs 14.18 VLVH-Fc were cloned into pQF62 and generated pQF204 and pQF205, respectively. Five different lengths of protein domains were used in this study: ch14.18 scFv VLVH (289 aa), ch14.18 scFv VHVL (289 aa), CD8 α molecule hinge (62 aa, a flexible spacer)^52^, a 13 aa rigid linker (EAEAAAKEAAAKA)^53^, and a 20 aa flexible (G4S)4^53^. Figure 1 is a schematic representation of the gD insertion mutants and deletion mutants generated by PCR used in the current studies. The amino acids of HSV-1 gD were numbered after the putative signal peptide cleavage.

We used a two-step red-mediated recombination strategy^54^,^55^ to make the bacterial artificial chromosome (BAC) viruses based on pGS3217 (kindly provided by Dr. Gregory Smith, Northwestern University). GS3217 is an HSV-1 F strain that carries the red fluorescence protein (RFP) tdTomato reporter gene with a nuclear localization signal under the control of a CMV promoter. GS3217 was derived from a BAC kindly provided by Dr. Yasushi Kawaguchi from the University of Toyko^56^. The BACs were transfected into neuroblastoma cells and VD60 cells in order to generate viruses. Plasmids and BACs made for this study were sequenced by the Northwestern Genomic core facility.

### Western blots

Whole cell lysates were examined by Western blot to assess the expression of the gD mutants. CHO-K1 cells were seeded in 6-well plates and transfected with 1.5 μg of plasmids expressing FLAG-tagged gD mutants or an empty vector using 5 μl of Lipofectamine 2000. After 24 h, the cells were detached using versene (0.2 g EDTA/liter in PBS), rinsed with PBS, and lysed in 200 μl of lysis buffer (25 mM Tris-HCl, pH 7.4, 150 mM NaCl, 5 mM EDTA, 10 mM NaF, 1 mM Na_3_VO_3_, 1% Nonidet P-40) containing protease inhibitors (Roche Diagnostics, Indianapolis, IN). After boiling for 5 min under reducing conditions, proteins were separated by SDS-PAGE on 4–20% gels and blotted. Blots were probed with rabbit anti-FLAG antibodies (Sigma, F7425) at a 1:1000 dilution for 1 h at room temperature, followed by anti-rabbit secondary antibody coupled to horseradish peroxidase (HRP) and ECL™ detection reagents (GE Healthcare).

### CELISA

Cell-based ELISA (CELISA) was used to evaluate the cell surface expression of the FLAG-tagged glycoproteins. CHO-K1 cells were seeded in 96-well plates and transfected with 60 ng of plasmids encoding a FLAG-tagged gD mutant or empty vector using 0.15 μl of Lipofectamine 2000 (Invitrogen) in Opti-MEM (Invitrogen). After 24 h, the cells were rinsed and CELISA was performed as previously described^33^, using the anti-FLAG MAb (Sigma F1084), followed by biotinylated goat anti-mouse IgG (Sigma), streptavidin-HRP (GE Healthcare), and HRP substrate (BioFX).

### Cell fusion assay

A quantitative luciferase-based assay was used to determine cell-cell fusion activity of the glycoproteins^50^. Effector CHO-K1 cells were seeded overnight in 6-well plates and then transfected with 400 ng each of plasmids encoding T7 RNA polymerase, gB or mutant gB, gH, gL, F-gD or a mutant gD, or with different combinations of these glycoproteins, using 5 μl of Lipofectamine 2000. Target CHO-K1 cells were seeded overnight in 6-well plates and then transfected with a plasmid of luciferase under the control of T7 promoter and a plasmid encoding PILRα, nectin-1 or HVEM using 5 μL of Lipofectamine 2000. Six h after transfection, the cells were detached with versene, and resuspended in 1.5 ml of F12 medium supplemented with 10% FBS. Effector and target cells were mixed in a 1:1 ratio and replated in 96-well plates. After 18 h, luciferase activity was determined using lysis buffer and luciferase substrate (Promega) and a Wallac-Victor luminometer (Perkin Elmer).

### Preparation of ch14.18 scFv-gD soluble protein and binding to neuroblastoma cells by flow cytometry

Supernatants from transfected cells were prepared as previously described^35^. Plates of 293 cells were transfected with pQF62, pQF204, pQF205, pQF199 and pQF201 using 293 Fectin (Invitrogen). The Fc concentration in the supernatants was normalized according to the Fc concentration determined by Western blotting. Neuroblastoma cells SK-N-Be (2) cells were stained with the supernatants (as the primary antibodies). The cells were stained with the secondary anti-human IgG FITC and analyzed with Flow Jo using data collected with an LSRII.

## Additional Information

**How to cite this article**: Fan, Q. *et al*. Mapping sites of herpes simplex virus type 1 glycoprotein D that permit insertions and impact gD and gB receptors usage. *Sci. Rep.* **7**, 43712; doi: 10.1038/srep43712 (2017).

**Publisher's note:** Springer Nature remains neutral with regard to jurisdictional claims in published maps and institutional affiliations.

## Figures and Tables

**Table 1:** Plasmids generated for this study.

Construct	Comment
pQF185	K1-P32 in PDM80 was replaced by 14.18 scfv VHVL (G4S)4
pQF187	K1-P32 in PDM80 was replaced by 14.18 scfv VLVH (G4S)4
pQF199	The PCR product of the ectodomain of pQF185 was cloned into pQF62
pQF201	The PCR product of the ectodomain of pQF187 was cloned into pQF62
pQF204	The PCR product of the ectodomain of pdCs 14.18 VHVL-Fc was cloned into pQF62
pQF205	The PCR product of the ectodomain of pdCs 14.18 VLVH-Fc was cloned into pQF62
pQF233	14.18 scfv VHVL (G4S)4 was inserted after A12 of pQF113
pQF234	14.18 scfv VHVL (G4S)4 was inserted after V34 of pQF169
pQF235	14.18 scfv VLVH (G4S)4 was inserted after A12 of pQF113
pQF236	14.18 scfv VLVH (G4S)4 was inserted after V34 of pQF169
pQF237	14.18 scfv VHVL (G4S)4 was added before G33 in pQF170
pQF238	14.18 scfv VLVH (G4S)4 was added before G33 in pQF170
pQF239	(G4S)4 was inserted after G187 in pQF169
pQF240	Rigid linker EAEAAAKEAAAKA was inserted after G187 in pQF169
pQF241	CD8 alpha hinge was inserted after G187 in pQF169
pQF242	14.18 scfv VLVH (G4S)4 was inserted after G187 in pQF169
pQF243	(G4S)4 was inserted after G243 in pQF169
pQF244	Rigid linker EAEAAAKEAAAKA was inserted after G243 in pQF169
pQF245	CD8 alpha hinge was inserted after G243 in pQF169
pQF246	14.18 scfv VLVH (G4S)4 was inserted after G243 in pQF169
pQF247	(G4S)4 was inserted after T260 in pQF169
pQF248	Rigid linker EAEAAAKEAAAKA was inserted after T260 in pQF169
pQF249	CD8 alpha hinge was inserted after T260 in pQF169
pQF250	14.18 scfv VLVH (G4S)4 was inserted after T260 in pQF169
